# Paradoxical Fat Embolism Syndrome During Total Hip Arthroplasty Without a Patent Foramen Ovale: A Case Report With Transesophageal Echocardiographic Findings

**DOI:** 10.7759/cureus.103556

**Published:** 2026-02-13

**Authors:** Tokimitsu Hibino, Yusuke Okui, Takuya Fujimoto, Yoshie Toba

**Affiliations:** 1 Department of Anaesthesiology, Seirei Hamamatsu General Hospital, Hamamatsu, JPN; 2 Department of Orthopaedic Surgery, Seirei Hamamatsu General Hospital, Hamamatsu, JPN

**Keywords:** cementless total hip arthroplasty, fat embolism syndrome (fes), paradoxical brain embolism, point-of-care ultrasonography, pulseless electric activity, transesophageal echocardiography (tee)

## Abstract

Fat embolism syndrome (FES) is a severe orthopedic complication leading to respiratory failure and neurological impairment. While bone marrow or fat entry into venous circulation is common during total hip arthroplasty (THA), cardiac arrest rarely results.

An 82-year-old woman developed pulseless electrical activity (PEA) during elective THA. During femoral reaming, a sudden decline in peripheral oxygen saturation (SpO_2_: 100% to 89%) and end-tidal carbon dioxide (EtCO_2_: 28 to 12 mmHg) was observed, followed by precipitous circulatory collapse. Transesophageal echocardiography (TEE) captured real-time video of numerous microemboli in all cardiac chambers, confirming pulmonary and paradoxical embolism. Despite successful resuscitation and surgical termination, TEE showed persistent embolic flow from the inferior vena cava (IVC). Postoperatively, the patient remained unconscious due to extensive cerebral infarction, resulting in permanent neurological sequelae.

Fat embolism during THA can be fatal. This case, documented via intraoperative imaging, highlights the need for rapid point-of-care diagnosis and proactive surgical techniques to minimize intramedullary pressure.

## Introduction

Fat embolism syndrome (FES) is a potentially fatal complication of long bone fractures or orthopedic procedures, such as hip or knee arthroplasty, where bone marrow components enter the pulmonary and systemic circulation, leading to respiratory failure, impaired consciousness, and petechiae [1]. While symptomatic fat embolism associated with trauma is well-documented, severe cases leading to cardiac arrest during elective total hip arthroplasty (THA) are rare [2].

We report a case of pulseless electrical activity (PEA) that occurred during elective THA. Intraoperative transesophageal echocardiography (TEE) enabled real-time detection of massive fat emboli, revealing not only pulmonary obstruction but also paradoxical systemic embolism. This phenomenon can occur even without a patent foramen ovale as fat emboli deform and traverse the pulmonary capillary bed [2]. Although prompt diagnosis allowed for immediate surgical termination and initial resuscitation, extensive cerebral infarction resulted in irreversible neurological sequelae. This report presents TEE findings of systemic embolism, accompanied by video footage, and discusses the diagnostic challenges and potential preventive strategies for intraoperative fat embolism based on a literature review.

## Case presentation

An 82-year-old woman (height: 153 cm, weight: 62 kg) presented with right hip pain and was scheduled for right THA due to osteoarthritis. Her medical history included hypertension, dyslipidemia, and a previous cerebral infarction. Five years prior, she had undergone a left THA without complications. At our facility, THA is typically performed under general anesthesia combined with epidural anesthesia. However, in this case, we opted against epidural anesthesia to facilitate the early resumption of postoperative anticoagulant therapy. Instead, spinal anesthesia was selected for intraoperative and acute postoperative pain control.

**Figure 1:**
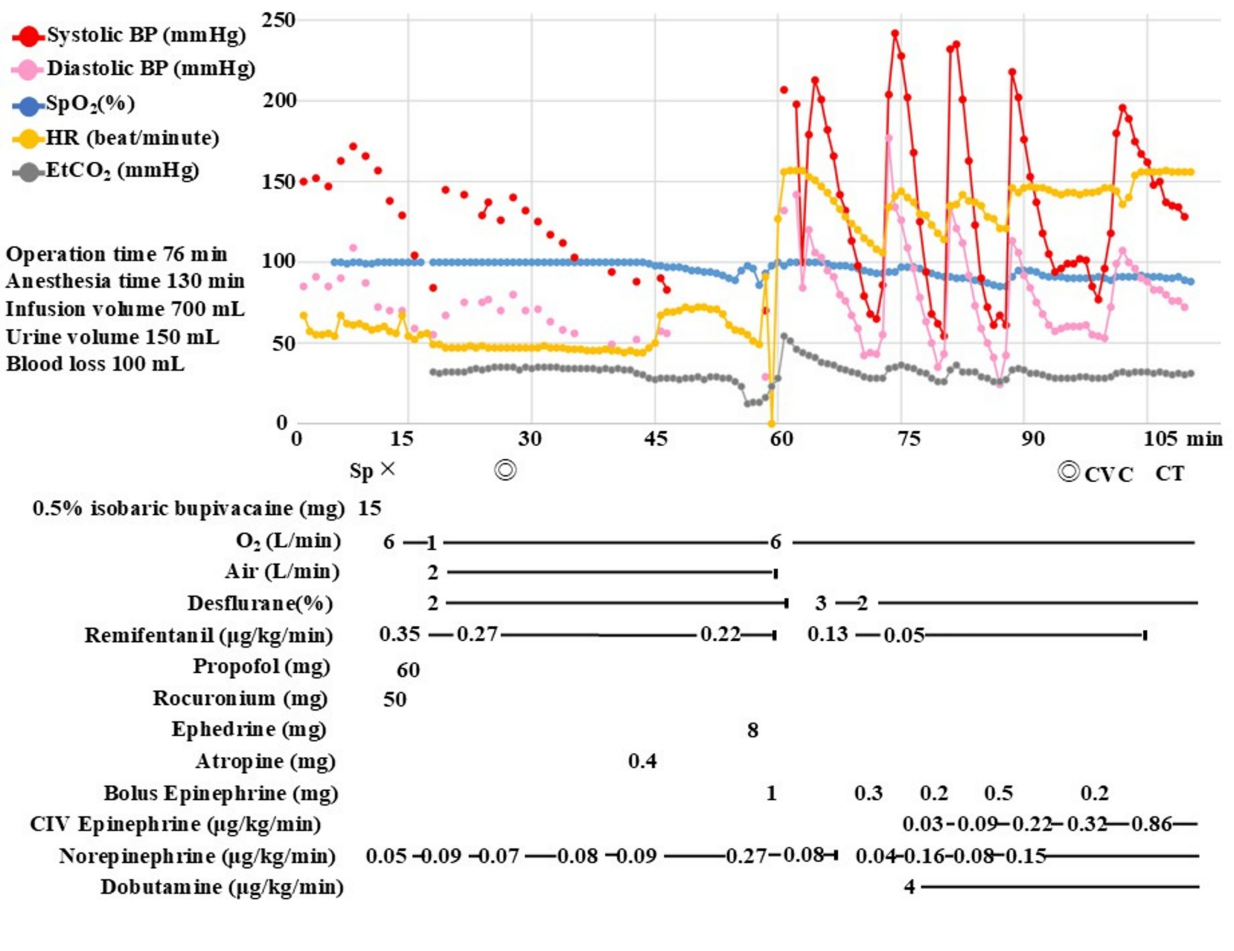
Anesthesia record highlighting the timeline of clinical deterioration Abbreviations: BP, blood pressure; CIV, continuous intravenous infusion; CT, computed tomography; CVC, central venous catheter; EtCO_2_, end-tidal carbon dioxide; HR, heart rate; PEA, pulseless electrical activity; SpO_2_, peripheral oxygen saturation Symbols and markers: Sp, spinal anesthesia start; ×, general anesthesia start; ◎, operation start/end; PEA, onset of PEA; CVC, CVC insertion; CT, transport for CT imaging The x-axis represents the time elapsed from the induction of anesthesia, while the y-axis indicates blood pressure, heart rate, SpO_2_, and EtCO_2_ values. Notably, a gradual decline in both SpO_2_ and EtCO_2_ preceded the onset of PEA by 15 minutes. Following the acute circulatory collapse, blood pressure became profoundly unstable, initially requiring frequent boluses of epinephrine. Subsequent hemodynamic stabilization was only achieved through high-dose continuous epinephrine infusion.

**Figure 2:**
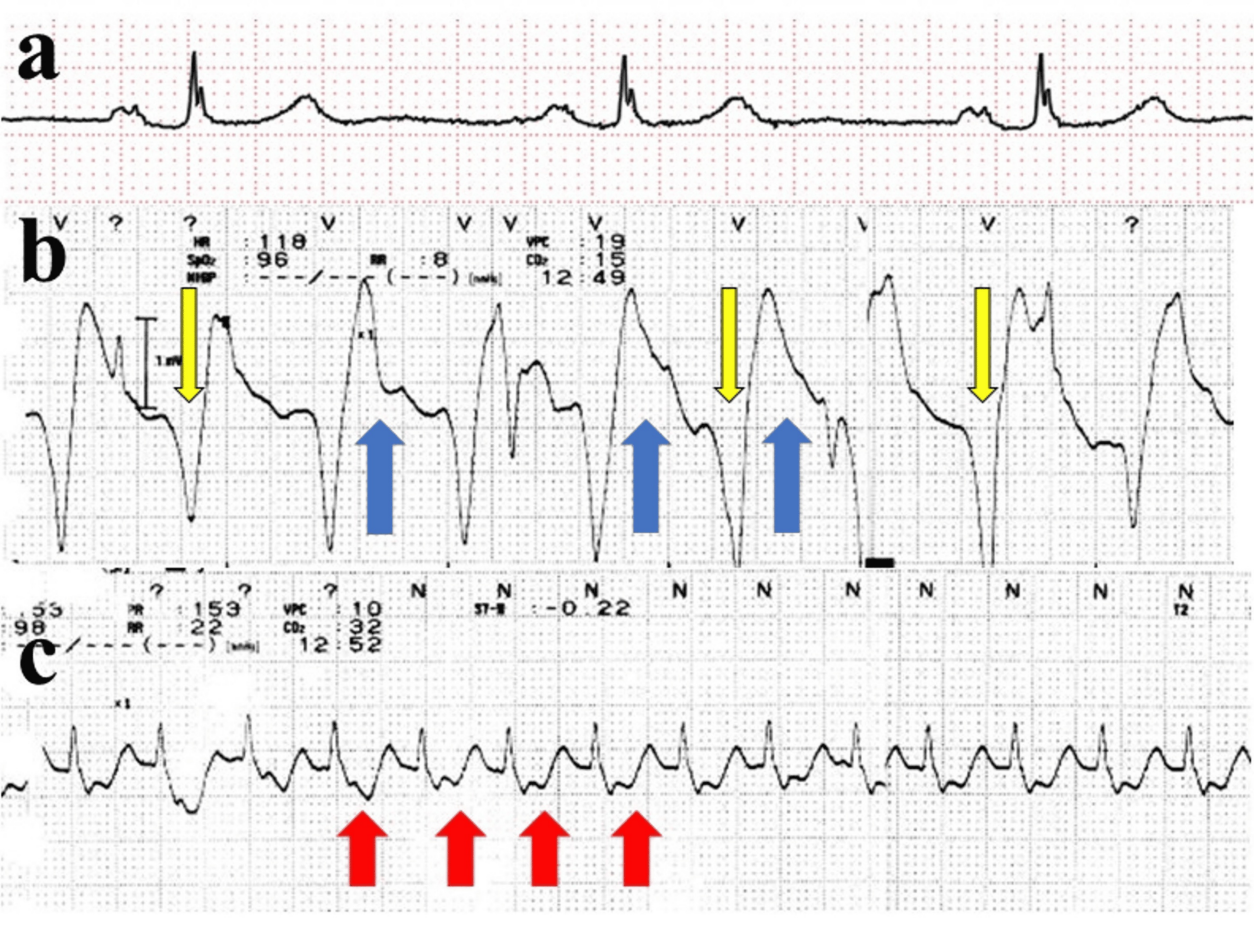
Sequential ECG changes (lead II) Abbreviations: AV, atrioventricular; ECG, electrocardiogram; PEA, pulseless electrical activity; ROSC, return of spontaneous circulation (a) Preoperative ECG showing first-degree AV block, nonspecific T-wave abnormalities, and bradycardia. (b) ECG during PEA demonstrating widened QRS complexes (yellow arrow), ST-segment elevation (blue arrow), and tachycardia. (c) ECG after ROSC showing narrowed QRS complexes and persistent tachycardia with new-onset ST-segment depression (red arrow).

Upon entering the operating room, standard monitoring was established, including electrocardiography, non-invasive blood pressure, peripheral oxygen saturation (SpO_2_), and body temperature. Spinal anesthesia was performed at the L2/L3 level via a paramedian approach using 15 mg of 0.5% isobaric bupivacaine. Given the patient’s age and the anticipated hypotension from combined spinal and general anesthesia, a norepinephrine infusion was initiated at 0.05 μg/kg/minute at induction. General anesthesia was induced with propofol, remifentanil, and rocuronium, and maintained with desflurane and remifentanil. The surgical procedure was performed according to standard protocols without any immediate surgical complications or excessive blood loss. Twenty-two minutes after the start of surgery, while the procedure was progressing uneventfully, 0.4 mg of atropine was administered to treat bradycardia (44 beats/minute). Although the heart rate increased to 70 beats/minute and blood pressure rose from 88/52 to 92/57 mmHg, SpO_2_ began a gradual decline from 100%. This was initially suspected to be a measurement error due to peripheral coldness, and the forced-air warmer was adjusted from 40°C to 46°C. Recurrent blood pressure measurement errors were initially attributed to premature atrial contractions; however, as the inability to measure blood pressure persisted for over five minutes, an arterial line was prepared. At minute 37, during femoral reaming, SpO_2_ dropped to 89%, and end-tidal carbon dioxide (EtCO_2_) rapidly decreased from 28 to 12 mmHg (Figure 1). Despite the lack of any adverse intraoperative surgical events up to this point, PEA was diagnosed based on the loss of SpO_2_ waveforms and the absence of palpable carotid pulses (Figure 2).

Cardiopulmonary resuscitation was immediately initiated, and spontaneous circulation was restored after 1 mg epinephrine administration. TEE was performed to identify the cause of the collapse. It revealed hyperechoic microparticles in the four cardiac chambers, pulmonary artery, and inferior vena cava (Videos 1-3). The atrial septum was bowed toward the left atrium, and the right ventricle was dilated, compressing the left ventricle (Videos 1, 4).

**Video 1:** Mid-esophageal five-chamber view of the heart during the embolic event This video depicts a mid-esophageal five-chamber view; however, reverberation artifact from the junction of the left pulmonary vein and left atrium obscures the visualization of the left ventricle. The red arrow indicates the atrial septum bowing into the left atrium, reflecting right-sided pressure overload. Heterogeneous hyperechoic microparticles are visible within the right atrium, right ventricle, and left atrium. Notably, microparticles can be observed flowing from the left pulmonary vein into the left atrium. Although a massive quantity of microparticles is present in the right heart, a smaller but distinct amount is also evident in the left atrium. Despite the septal displacement toward the left atrium, no microparticles are seen crossing the septum via a patent foramen ovale, suggesting the particles reached the left-sided circulation via the pulmonary vasculature.

**Video 2:** Mid-esophageal aortic short-axis view during the embolic event The blue arrow indicates the main pulmonary artery, which is filled with numerous microparticles. Notably, a smaller yet distinct number of microparticles is also visible within the aorta. This finding further confirms that the embolic material had entered the systemic arterial circulation.

**Video 3:** Transgastric longitudinal view of the inferior vena cava Abbreviations: IVC, inferior vena cava Heterogeneous hyperechoic microparticles, ranging from less than 1 mm to 3 mm in size, are observed flowing from the distal IVC (yellow arrow) toward the right atrium. Although this video was recorded immediately following the acute circulatory deterioration, embolic flow within the IVC persisted for 20 minutes after the cessation of bone manipulation.

**Video 4:** Transgastric short-axis view of the left ventricle at the basal level This slightly oblique slice demonstrates marked right-sided pressure overload. The interventricular septum (white arrow) protrudes toward the left ventricle, resulting in a characteristic “D-shaped” appearance. This septal shift severely reduces left ventricle end-diastolic volume and impairs cardiac output, consistent with the observed hemodynamic instability.

Despite no visible interatrial communication, a diagnosis of pulmonary and paradoxical embolism (air or fat) was made. The surgery was immediately terminated; the surgeons closed the incision with the trial prosthesis remaining in the femur. Notably, TEE continued to show microparticles flowing from the inferior vena cava into the right atrium even after skin closure was completed. Blood tests revealed a prothrombotic state (D-dimer: 42.7 μg/mL, fibrin/fibrinogen degradation products: 250.4 μg/mL; Table 1). Due to persistent circulatory instability, bolus epinephrine was required every six minutes (Figure 1). Following a contrast-enhanced computed tomography scan, the patient was transferred to the intensive care unit (ICU).

**Table 1:** Longitudinal laboratory data and clinical trends Abbreviations: BE, base excess; D, day; EtCO_2_, end-tidal carbon dioxide; FDP, fibrin/fibrinogen degradation products; fg, fibrinogen; FiO_2_, fraction of inspired oxygen; Hb, hemoglobin; HCO3-, bicarbonate; h, hours; PaCO_2_, partial pressure of arterial carbon dioxide; ICU, intensive care unit; PaO_2_, partial pressure of arterial oxygen; Plt, platelet count Summary: The data illustrate the progression from a catastrophic intraoperative metabolic crisis to steady physiological recovery. Key findings include an immediate, profound metabolic acidosis (pH nadir) during the embolic event, followed by a delayed peak in lactate and D-dimer during the early ICU phase, with subsequent stabilization over the first eight postoperative days. Technical notes: †Excluding the “Event” data, EtCO_2_ measurements were performed in the ICU. The EtCO_2_ sensors were positioned near the ventilator unit rather than at the patient’s airway, resulting in systematically lower readings; ventilation was managed primarily based on PaCO_2_ levels. Acid-base status: pH reached its nadir during the intraoperative crisis and normalized within 12 hours of ICU admission. In contrast, lactate levels peaked six hours after ICU admission and subsequently declined. Coagulation profile: D-dimer levels peaked two hours after ICU admission; while they decreased thereafter, levels remained elevated (>10 μg/mL) through postoperative day 8. FDP was markedly elevated (250.4 μg/mL) during the intraoperative crisis. Reference ranges: Based on the standards of the clinical laboratory at Seirei Hamamatsu General Hospital, Hamamatsu, Japan.

	Day -41	Preoperative	Event	2 hours	4 hours	6 hours	9 hours	12 hours	17 hours	22 hours	24 hours	30 hours	35 hours	2 days	3 days	4 days	5 days	6 days	8 days	Reference range
pH	-	-	7.214	7.223	7.225	7.304	7.345	7.41	7.43	7.458	7.45	7.464	7.477	7.477	7.46	7.473	7.475	7.444	7.456	7.375±0.025
PaO_2 _(mmHg)/FiO_2_	-	-	173/1.0	98.6/0.5	104/0.5	52.1/0.5	103/0.5	114/0.5	123/0.5	81/0.4	89/0.4	81.1/0.4	84.5/0.4	87.4/0.4	91.4/0.4	76.4/0.4	69.7/0.4	76.3/0.35	88.5/0.35	90±10
EtCO_2_ (mmHg) †	-	-	38	-	28	14	18	20	19	17	22	20	23	22	22	26	27	30	32	40±5
HCO_3_^-^ (mmol)	-	-	24.6	18.3	18.3	16.8	18	18.9	20.1	18.9	19.6	19.6	21	23	23.5	27.1	28.7	30.1	30.6	24±2
BE (mmol)	-	-	-4.1	-8.9	-8.9	-8.7	-6.8	-4.9	-3.4	-4.2	-3.7	-3.5	-2	-0.3	1	3.2	4.7	5.5	6.1	0±2
Lactate (mg/dL)	-	-	26	32	29	44	37	25	26	18	17	19	17	14	14	16	18	12	9	10±6
Hb (g/dL)	13	11.5	11.9	10.2	-	-	10.7	-	9.4	-	-	-	-	8	7.1	6.7	7.7	7.5	7.5	13.2±1.6
Plt (× 10⁹/L)	156	174	146	152	-	-	98	-	74	-	-	-	-	89	79	86	112	111	167	253±95
D dimmer (μg/mL)	0.6	-	42.7	168.6	-	-	-	-	42.7	-	-	-	-	21.7	9	13.1	19.3	15.9	10.2	<1.0
FDP (μg/mL)	-	-	250.4	-	-	-	-	-	-	-	-	-	-	-	-	-	-	-	-	<5.0
fg (mg/dL)	-	-	347	191	-	-	160	-	237	-	-	-	-	367	393	367	290	264	381	300±100
AT(%)	-	-	71	-	-	-	-	-	-	-	-	-	-	-	-	-	-	-	-	105±25

Hemodynamics gradually stabilized, and epinephrine was discontinued five hours after ICU admission. Norepinephrine and dobutamine were discontinued on postoperative days 2 and 3, respectively. Regarding laboratory findings upon ICU admission, metabolic acidosis and markedly elevated D-dimer levels were observed. While the pH normalized within 12 hours of admission and D-dimer levels gradually decreased, the latter remained above 10 μg/mL even on postoperative day 8 (Table 1).

**Figure 3:**
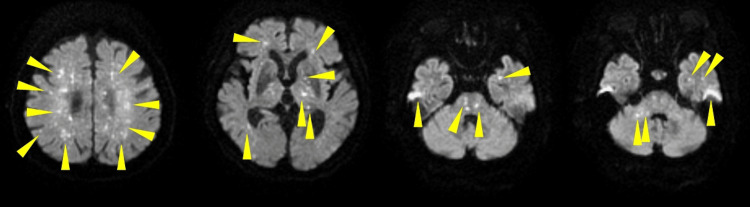
Brain MRI (diffusion-weighted imaging) on postoperative day 4 Abbreviations: MRI, magnetic resonance imaging Axial slices, arranged from cranial to caudal, demonstrate extensive high-intensity foci consistent with a “starfield pattern.” These lesions are scattered across multiple regions, including the frontal, temporal, and occipital lobes, the limbic system, the cerebellum, and the brainstem (representative lesions indicated by yellow arrowheads). The imaging was obtained to investigate persistent impaired consciousness following the surgery.

Despite this hemodynamic stabilization, the patient’s consciousness remained severely impaired (Glasgow Coma Scale E1VTM3). Magnetic resonance imaging performed on postoperative day 4 revealed multiple cerebral infarcts (Figure 3), and petechiae were observed on the right palpebral conjunctiva; these findings led to a definitive diagnosis of fat embolism syndrome.

A tracheostomy was performed on postoperative day 8 due to prolonged respiratory failure. Although the patient was transferred from the ICU to a general ward on postoperative day 13, her recovery was severely hindered by her advanced age and persistent unconsciousness (Glasgow Coma Scale E1VTM3). This clinical state led to a cycle of recurrent complications, including intermittent wound, urinary tract, and respiratory infections, requiring multiple courses of antibiotic therapy and intensive bedside care. In accordance with Japanese clinical pathways for patients in a persistent vegetative state, transfer to a long-term care facility is only feasible once complete systemic and infectious stability is achieved. Consequently, after 161 days of acute and sub-acute management to ensure her stability, she was finally transferred to a skilled nursing facility.

Written informed consent for the publication of this case report was obtained from the patient’s family due to the patient’s persistent unconsciousness. This is supported by the patient’s own pre-procedural consent, which authorized the use of clinical data for educational purposes and the reporting of rare complications.

## Discussion

Diagnostic considerations

This patient experienced an extensive cerebral infarction due to intraoperative systemic fat embolism. When her condition acutely deteriorated, fat and air embolism were the primary differential diagnoses, given the concurrent femoral reaming and rapid decline in SpO_2_ and EtCO_2_. Although TEE confirmed pulmonary and systemic embolism via microparticles in both cardiac chambers alongside right heart overload, identifying the specific embolic agent intraoperatively was challenging. Retrospectively, the detected hyperechoic microparticles showed heterogeneous echogenicity and varied in size (less than 1-3 mm). In contrast, air possesses significantly lower acoustic impedance than biological tissue, reflecting nearly 100% of ultrasound waves [3]. Consequently, air typically produces uniform hyperechoic echoes with distinct acoustic shadowing, distinguishing it from fat. However, in the clinical setting, the irregular and rapid movement of numerous microparticles made immediate differentiation difficult, requiring clinicians to prioritize hemodynamic stabilization while interpreting TEE findings.

The reported complications of THA include cardiac arrest (0.6%-10%) [4], myocardial infarction (0.4%), pulmonary embolism (0.7%), deep vein thrombosis (1.5%), and mortality (0.5%) [5]. Subclinical or mild fat embolism occurs in up to 49% of cases [6], while air embolism is reported in 57% [7]. Given these similar frequencies, epidemiological differentiation is unreliable. However, animal studies by Wang et al. suggest that fat has a lower lethal dose than air and is more frequently associated with paradoxical embolism [8]. Furthermore, fat emboli can deform to traverse the pulmonary microcirculation and reach the left heart, suggesting that a patent foramen ovale may not be a prerequisite for paradoxical fat embolism [9,10]. Thus, identifying the embolic agent and characterizing its flow patterns are crucial for prognosis, necessitating a careful, multi-physician review of TEE findings.

According to a recent review [11], TEE has been increasingly recognized as a valuable tool for monitoring embolic material during orthopedic surgery, despite the evolving evidence for its routine use. Specifically, small prospective studies cited in the review suggest that the detection of large emboli (>10 mm) via TEE correlates with an increased incidence of FES and mortality [11]. While our case primarily involved a massive shower of smaller microparticles (less than 1-3 mm) rather than a single large mass, the continuous and high-volume nature of the embolic flow we observed suggests that TEE may help clinicians identify surgical phases associated with significant embolic increases.

Prevention of catastrophic outcomes and early warning signs

The classic triad of fat embolism syndrome includes respiratory failure, impaired consciousness, and petechiae, which form the basis of Gurd’s and Schonfeld’s diagnostic criteria [12]. A retrospective review of the anesthesia record revealed that SpO_2_ and EtCO_2_ began to decline 15 minutes before the onset of PEA (Figure 1). Since acetabular reaming was already complete, invasion of the medullary cavity had likely initiated the embolic shower. Had transthoracic echocardiography detected these emboli at this stage, halting bone manipulation might have prevented circulatory collapse. However, TEE routinely detects embolic material in 94% of THA patients [13], whereas life-threatening fat embolism syndrome occurs in only about 0.1% [14]. This makes immediate surgical termination upon any embolic detection impractical.

Consequently, clinicians must establish thresholds for surgical interruption based on combined monitoring. We propose that a “persistent EtCO_2_ decline” (e.g., a gradual decrease of 5-10 mmHg from baseline despite stable ventilation) should be emphasized as a critical and actionable “early warning sign” of severe fat embolism. As pulmonary obstruction progresses and dead space increases, EtCO_2_ responds acutely. This case suggests that intervening when subtle monitor changes occur, such as intensifying intramedullary lavage or temporarily pausing the procedure to allow for hemodynamic stabilization and embolic clearance, may be the only opportunity to prevent catastrophic outcomes. Indeed, Visnjevac et al. reported that using changes in EtCO_2_ as an alert tool during therapeutic interventions for pulmonary embolism improves survival rates [15], supporting our proposal. Furthermore, we observed embolic flow in the inferior vena cava for 20 minutes after ceasing manipulation and through skin closure. This indicates that fat migration does not terminate immediately upon stopping the surgical stimulus. While early intervention might have prevented PEA by limiting the total embolic load, preventing the cerebral infarction itself might still have been difficult in this specific case. Therefore, preventing venous entry of fat is paramount. Inserting a prosthetic stem increases intramedullary pressure, driving fat into the circulation [16]. Singh et al. reported that venting the distal femur and applying suction during stem insertion can mitigate this pressure increase [17]. Since this case, our institution has implemented more rigorous intramedullary saline irrigation and is considering additional measures to minimize intramedullary pressure.

## Conclusions

We managed a case of circulatory collapse due to fat embolism during THA, complicated by extensive cerebral infarction via paradoxical embolism. TEE provided vital visualization of the embolic trajectory from the right to the left heart. Paradoxical fat embolism is a grave complication that can occur regardless of patent foramen ovale status, potentially via intrapulmonary shunts. To prevent such outcomes, establishing intervention protocols based on early warning signs, particularly sustained decreases in EtCO_2_ and characteristic echocardiographic findings, is warranted. Future large-scale studies are needed to evaluate the efficacy of surgical techniques, such as intramedullary pressure management, in reducing embolic load.

## References

[1] Akhtar S (2009). Fat embolism. Anesthesiol Clin.

[2] Kwon J, Coimbra R (2024). Fat embolism syndrome after trauma: what you need to know. J Trauma Acute Care Surg.

[3] Sommer FG, Taylor KJ (1980). Differentiation of acoustic shadowing due to calculi and gas collections. Radiology.

[4] Koessler MJ, Fabiani R, Hamer H, Pitto RP (2001). The clinical relevance of embolic events detected by transesophageal echocardiography during cemented total hip arthroplasty: a randomized clinical trial. Anesth Analg.

[5] Mantilla CB, Horlocker TT, Schroeder DR, Berry DJ, Brown DL (2002). Frequency of myocardial infarction, pulmonary embolism, deep venous thrombosis, and death following primary hip or knee arthroplasty. Anesthesiology.

[6] Kim YH, Oh SW, Kim JS (2002). Prevalence of fat embolism following bilateral simultaneous and unilateral total hip arthroplasty performed with or without cement : a prospective, randomized clinical study. J Bone Joint Surg Am.

[7] Spiess BD, Sloan MS, McCarthy RJ, Lubenow TR, Tuman KJ, Matz SD, Ivankovich AD (1988). The incidence of venous air embolism during total hip arthroplasty. J Clin Anesth.

[8] Wang AZ, Zhou M, Jiang W, Zhang WX (2008). The differences between venous air embolism and fat embolism in routine intraoperative monitoring methods, transesophageal echocardiography, and fatal volume in pigs. J Trauma.

[9] Nikolić S, Zivković V, Babić D, Djonić D, Djurić M (2012). Systemic fat embolism and the patent foramen ovale--a prospective autopsy study. Injury.

[10] Mano D, Campos P, Vale B, Pinto A (2021). Stroke and respiratory failure: mind the shunt!. Eur J Case Rep Intern Med.

[11] Stump B, Weinhouse G (2016). Fat embolism syndrome: fact or myth?. Curr Trauma Rep.

[12] Rothberg DL, Makarewich CA (2019). Fat embolism and fat embolism syndrome. J Am Acad Orthop Surg.

[13] Pitto RP, Koessler M, Draenert K (1998). The John Charnley Award. Prophylaxis of fat and bone marrow embolism in cemented total hip arthroplasty. Clin Orthop Relat Res.

[14] Freedman KB, Brookenthal KR, Fitzgerald RH Jr, Williams S, Lonner JH (2000). A meta-analysis of thromboembolic prophylaxis following elective total hip arthroplasty. J Bone Joint Surg Am.

[15] Visnjevac O, Pourafkari L, Nader ND (2014). Role of perioperative monitoring in diagnosis of massive intraoperative cardiopulmonary embolism. J Cardiovasc Thorac Res.

[16] Pitto RP, Hamer H, Fabiani R, Radespiel-Troeger M, Koessler M (2002). Prophylaxis against fat and bone-marrow embolism during total hip arthroplasty reduces the incidence of postoperative deep-vein thrombosis: a controlled, randomized clinical trial. J Bone Joint Surg Am.

[17] Singh VA, Sarrafan S, Veriah RS (2018). Distal medullary canal decompression in long stem hip replacement in long bone metastasis: does it reduce cardiopulmonary complications?. Indian J Orthop.

